# Abiotic and biotic factors controlling fine root biomass, carbon and nutrients in closed-canopy hybrid poplar stands on post-agricultural land

**DOI:** 10.1038/s41598-019-42709-6

**Published:** 2019-04-18

**Authors:** Julien Fortier, Benoit Truax, Daniel Gagnon, France Lambert

**Affiliations:** 1 Fiducie de recherche sur la forêt des Cantons-de-l’Est/Eastern Townships Forest Research Trust, 1 rue Principale, Saint-Benoît-du-Lac, Qc J0B 2M0 Canada; 2 Department of Biology, University of Regina, 3737 Wascana Parkway, Regina, Sk S4S 0A2 Canada

**Keywords:** Forestry, Forest ecology

## Abstract

Fine roots (diameter <2 mm) have a pivotal role in resource acquisition, symbiosis development, and for elemental cycling in forests. Various abiotic and biotic factors affect their biomass and nutrient content. Understanding the effect of these factors on root traits could improve biogeochemical modelling, nutrient management and ecosystem services provision in planted forests. Data from 14-year old poplars planted along a fertility/climatic gradient in Southeastern Canada, show that live fine root biomass varied with genotype and environment, was negatively correlated to soil fertility, and uncorrelated to tree size. Dead fine root biomass varied with genotype and peaked during fall and in colder environments with slower element cycling. Root chemistry also varied with environment, genotype and season. The genotype producing recalcitrant leaf litter had the highest root biomass, suggesting a compensation strategy. Along the studied gradient, plasticity level observed for some root traits (biomass, element contents) was genotype-specific and high for some genotypes. Regionally, such plasticity patterns should be considered in elemental budgets, for nutrient management and ecosystem services provision in plantations (carbon storage, nutrient retention). The small inter-site aboveground productivity differences observed suggest that plasticity in fine root growth may contribute to overcome nutrient limitations on less fertile marginal lands.

## Introduction

Worldwide, approximately 8.6 million ha are planted with fast-growing poplars for timber production and environmental protection^1^. Poplar afforestation can also reduce carbon dioxide (CO_2_) in the atmosphere by promoting carbon storage in plant biomass and, under certain conditions, in soil^2,3^. To avoid competition with food crops, poplars are increasingly planted on abandoned farmland, but regionally, these sites often have unequal soil fertility^4–6^.

Soil fertility and/or regional climate (or site elevation) gradients have a large effect on the aboveground biomass growth of hybrid poplars from different parental species^6,7^. However, limited and inconsistent information exists about the effect of soil fertility, and of other environmental and genetic factors, on fine root biomass of planted poplars^8^. Fine roots (i.e. root with a diameter <2 mm) are of great importance for the acquisition of soil nutrients and water, which limit plant growth^9^. While they represent only a minor fraction of poplar tree biomass^10^, fine roots have a pivotal role in the cycling of carbon (C) and nutrients because they are short-lived and nutrient-rich^9,11^. Nutrients released from fine root decomposition sometimes exceed the amount of nutrients released during leaf litter decay and an important proportion of the net primary productivity is allocated to fine roots^12,13^. Fine roots also form the network upon which mycorrhizal associations develop, improving tree nutrition, stress tolerance and disease protection^14^. Furthermore, some ecosystem services provided by tree plantations are linked to fine roots. For example, carbon inputs derived from poplar fine roots play a critical role in soil C sequestration following afforestation^15,16^. A better quantification of the nutrient and C pools located in poplar plantation fine roots is thus needed to improve biogeochemical modelling, long-term nutrient management and ecosystem services quantification in fast-growing plantations.

Several abiotic and biotic factors can affect fine root biomass and its nutrient concentrations and contents. In forests, these root traits vary widely between tree species and functional groups^9,13,17,18^. In boreal forests, fine root biomass was positively associated to mean annual temperature (MAT) and precipitation (MAP), and stand age, but was negatively related to soil fertility^13^. In temperate forests, fine root biomass increased with site elevation (or decreased with MAT)^19^. Yet, across different forest biomes, mean basal area and nutrients in leaf litter best predicted fine root biomass^17,20^. In the Scandinavian boreal forest, stand basal area was the strongest factor predicting fine root biomass^18^. Nutrient concentrations and contents in fine roots were also related to climate and soil nutrients^13,21^. Hence, it is unclear whether soil fertility, climatic or stand variables best predict fine root biomass or nutrients^22^. Moreover, factors related to fine root biomass in global or biome scale studies are not necessarily reflected in regional scale studies. For example, little variation in fine root biomass was observed along a steep gradient of aboveground biomass productivity and soil resource availability in longleaf pine (*Pinus palustris*) forests^23^.

In fast-growing poplars, few regional studies have evaluated the effect of abiotic and biotic factors on fine root biomass, nutrient concentrations and contents. Previous studies have shown that these traits are affected by genetic, environmental, seasonal and morphological factors. Wide variations in fine root biomass and in its plasticity level were observed between poplar genotypes^24–28^. Poplar species and hybrids producing more recalcitrant leaf litter (i.e. with high condensed tannin concentrations) also produced more fine roots; possibly to compensate for the negative feedback of leaf litter on soil N mineralization^29^. Allometric relationships between root biomass and aboveground traits have also been reported in planted and natural poplar stands^10,30–34^. However, allometric relationships between fine root biomass and aboveground traits were mostly observed in younger plantations^10,25^, probably because fine root biomass of many species only increases until canopy closure, and afterward remains constant and uncoupled with aboveground growth^18^.

In old-field environments, total coarse root biomass of poplars varied little across fertility gradients^30,35^. Yet, both positive and negative correlations between soil fertility and fine root biomass have been reported in *Populus*. Higher fine root biomass and N content have been observed in young *P*. *tremuloides* growing on soils with higher N availability^36^. Over a 6 year establishment-phase, Coleman and Aubrey^37^ found that increasing soil N and/or water availability led to subtle increases or to no change in *P*. *deltoides* fine root biomass, and concluded that stand developmental stage was the factor with overriding importance. Developmental stage also affected fine root biomass of a poplar short-rotation coppice, but a strong negative effect of N fertilization on fine root biomass appeared during the 4^th^ growing season^38^. A recent greenhouse study also showed that a low N supply changed gene expression, modified root architecture and led to an increase in fine root biomass, thus providing evidence of a unique nitrogen-adaptative mechanism regulating hybrid poplar root growth in response to soil N supply^39^. Yet, only a few field studies partly support this finding in older plantations^35,40,41^.

Fine root production and mortality rates also fluctuate during the growing season, with production and mortality peaks generally observed in the spring and fall, respectively^42–46^. However, seasonal evolution patterns in fine root mass may differ between hybrid types^24^. During the first year of growth, seasonal patterns in fine root N were also observed in different hybrid poplars, with N concentrations increasing towards the end of the growing season, as N resorbed from senesced leaves is stored in roots during the dormant season^26^.

In poplars, high nutrient availability in the soil is generally reflected by higher nutrient levels in foliage and in leaf litter^47–50^. However, evidence of a relationship between soil nutrient availability and fine root nutrient concentrations is limited within *Populus* species. Higher soil P was related to higher fine root P concentration in *P*. *tomentosa* plantations, but inconsistent trends where observed for fine root N and potassium (K)^51^. Yet, N-fertilization led to a 3-fold increase in fine root N concentrations in *P*. *tremuloides* clones^52^, which contradicts other field observations^36^. Given that both foliage and fine root nutrient concentrations can be affected by soil fertility, covariation between nutrient concentrations in foliage (green or senescent) and in fine roots is expected^53^. In addition, there are large variations in foliage and leaf litter chemistry between hybrid poplar genotypes from different parentages. Often, genotypes related to the *Aigeiros* section have higher nutrient concentrations in foliar tissues (green or senescent), than genotypes related to the balsam poplar (*Tacamahaca*) section^49,54,55^. However, such a trend was not observed for fine root nutrient concentrations^26^.

In this study, we evaluated the effect of genotype, environment and seasonality on fine root mass, nutrient concentrations and nutrient contents in 14 year-old hybrid poplar plantations with closed-canopies. We also evaluated if other abiotic and biotic factors (site elevation, soil properties, leaf litter chemistry and decay rate, tree size) were related to live fine root biomass, dead fine root biomass, and the nutrient content of both root compartments. We further evaluated covariation between nutrient concentrations in leaf tissues (green foliage and leaf litter) and in fine roots. The three plantations sites selected for this study where positioned along an edaphic and elevational (or climatic) gradient in the southern Québec region of Southeastern Canada. The three genotypes selected had different genetic assemblages between species from different sections: (1) genotype D × N-131 (hereafter named genotype D × N), a *P*. *deltoides* × *P*. *nigra* hybrid (synonym *P*. × *canadensis*); (2) genotype DN × M-915508 (hereafter named genotype DN × M), a *P*. × *canadensis* × *P*. *maximowiczii* hybrid and (3) genotype M × B-915311 (hereafter named genotype M × B), a *P*. *maximowiczii* × *P*. *balsamifera* hybrid.

The following hypotheses were tested: (1) an inverse relationship should be observed between soil fertility and live fine root biomass; (2) a compensatory response in fine root biomass should be observed for genotype DN × M, which produces low quality and slow decaying leaf litter^49^; (3) live fine root biomass should be higher in the spring, while dead fine root biomass should be higher in the fall; (4) the higher foliage and leaf litter nutrient concentrations of genotype D × N^49^, should be reflected in fine roots; and (5) nutrient concentrations in fine roots should be positively related to nutrient supply in the soil, and to nutrient concentrations in leaves (green foliage and leaf litter).

## Results

### Site and soil characteristics

All soil characteristics measured were significantly affected by the plantation environment (Table 1). Overall, the Brompton site, which is located at low elevation (170 m), benefited from the highest MAT and tended to be the most fertile (highest soil clay content, pH, base saturation, CEC, and supply rates of NO_3_, P, Ca and Mg; and lowest soil stone content, C:N ratio, and concentrations of C and organic matter). The soil of the La Patrie site (440 m of elevation) had the lowest pH and NO_3_ supply rate, but the highest NH_4_ supply rate. The soil of the Melbourne site (330 m of elevation) had the highest concentrations of organic matter, total C and total N, C:N ratio, K supply rate, but the lowest P supply rate.

**Table 1:** Site and soil characteristics of the three hybrid poplar plantation environments and characteristics of the studied genotypes (SE = Standard error of the mean) (modified from Fortier *et al*.^49^).

Site and soil characteristics	Brompton	Melbourne	La Patrie	SE	P-value
Site elevation (m)	170	330	440	—	—
Mean annual temperature (°C)	5.6	4.7	4.0	—	—
Mean total annual precipitation (mm/yr)	1146	1232	1370	—	—
Soil clay content (%)	24	14	16	—	—
Soil silt content (%)	49	37	47	—	—
Soil sand content (%)	27	49	37	—	—
Bulk density of fine earth fraction (g/cm^3^)	1.29	0.96	0.97	0.03	<0.0001
Soil stoniness (%)	0.3	5.6	11.6	1.0	<0.0001
Soil pH (water)	5.67	5.43	5.16	0.05	<0.0001
Soil organic matter (%)	4.60	6.93	4.82	0.24	<0.0001
Total soil C (mg/g)	21.5	33.9	24.5	1.7	0.0005
Total soil N (mg/g)	2.54	3.07	2.51	0.12	0.009
Soil C:N ratio	8.46	11.00	9.78	0.19	<0.0001
Soil base saturation (%)	47.9	26.3	30.2	2.4	<0.0001
Soil cation exchange capacity (meq/100 g)	14.6	12.6	11.4	0.7	0.01
Soil NO_3_ supply (µg/10 cm^2^/42d)	63.6	9.4	5.1	9.5	<0.0001
Soil NH_4_ supply (µg/10 cm^2^/42d)	4.22	5.06	6.82	0.31	0.0003
Mean soil NO_3_:NH_4_ ratio (molar basis)	4.38	0.54	0.22	—	—
Soil P supply (µg/10 cm^2^/42d)	5.37	1.23	3.59	0.75	0.007
Soil K supply (µg/10 cm^2^/42d)	18.9	41.3	25.7	5.8	0.05
Soil Ca supply (µg/10 cm^2^/42d)	2347	1863	2150	93	0.01
Soil Mg supply (µg/10 cm^2^/42d)	302	256	192	13	0.0002
Leaf litter mass remaining after 1 yr (% of initial mass)	19.6	38.4	60.6	3.9	<0.0001
Aboveground biomass of sampled trees (kg/tree)	149.6	134.7	136.8	8.2	0.41
Aboveground woody biomass yield (t/ha/yr)	7.69	6.07	6.54	0.57	0.17
**Hybrid poplar genotype characteristics**	**DN × M**	**D × N**	**M × B**	**SE**	**P-value**
Genotype number	915508	131	915311	—	—
Parental species (female)	*P*. × *canadensis*	*P*. *deltoides*	*P*. *maximowiczii*	—	—
Parental species (male)	*P*. *maximowiczii*	*P*. *nigra*	*P*. *balsamifera*	—	—
Leaf litter N concentration	0.68	1.23	0.83	0.04	<0.0001
Leaf litter P concentration	0.038	0.131	0.086	0.006	<0.0001
Leaf litter K concentration	0.34	1.06	0.32	0.08	<0.0001
Leaf litter Ca concentration	2.22	2.49	3.66	0.06	<0.0001
Leaf litter mass remaining after 1 yr (% of initial mass)	49.6	35.1	33.8	3.9	0.02
Aboveground biomass of sampled trees (kg/tree)	169.1	97.9	154.1	8.2	0.0001
Aboveground woody biomass yield (t/ha/yr)	7.58	4.58	8.14	0.57	0.002

Mean values of soil nutrient supply rates (across three sampling periods) were used in the ANOVA. Leaf litter nutrient concentration and woody biomass yield data were collected in the same experimental design the year preceding fine root sampling (after 13 growing seasons), while leaf litter mass remaining data were collected during the year of fine root sampling (14^th^ growing season)^49^.

**Figure 1:**
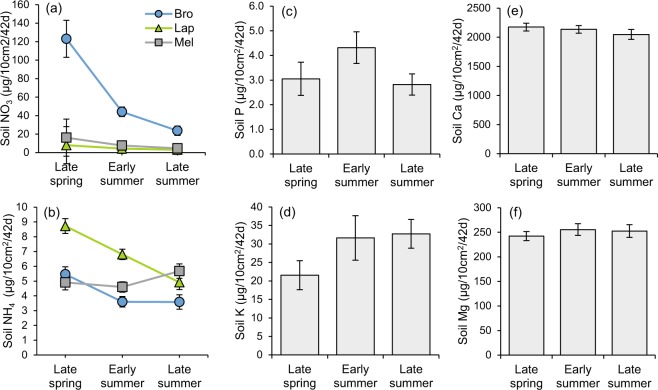
Season × Environment interaction effect on soil (**a**) NO_3_ and (**b**) NH_4_ supply rate and Season effect on soil (**c**) P, (**d**) K, (**e**) Ca and (**f**) Mg supply rate in hybrid poplar plantations. P-value of Season × Environment interaction effects are the following (according to MANOVA): NO_3_ (p = 0.05) and NH_4_ (p = 0.01). P-value of the Season effect are the following (according to MANOVA): NO_3_ (p < 0.0001), NH_4_ (p = 0.0005), P (p = 0.02), K (p = 0.004), Ca (p = 0.03), Mg (p = 0.03). Vertical bars are standard error of the mean.

There were significant seasonal fluctuations in soil nutrient supplies (Fig. 1). Soil NO_3_ declined at all sites through the growing season, but this decline was particularly large in magnitude at Brompton. NH_4_ supply also tended to decline through the growing season at Brompton and La Patrie. Conversely, soil K supply increased significantly through the growing season at all sites. Although the Season effect was significant for P, Ca and Mg supply rates, variations were relatively marginal.

### Aboveground biomass of sampled trees

There was no significant Environment effect (p = 0.41) on the aboveground woody biomass of trees selected for fine root sampling (Table 1). However, aboveground woody biomass significantly differed between genotypes (p = 0.0001), with woody biomass of genotype D × N being the lowest.

### Live and dead fine root biomass

**Figure 2:**
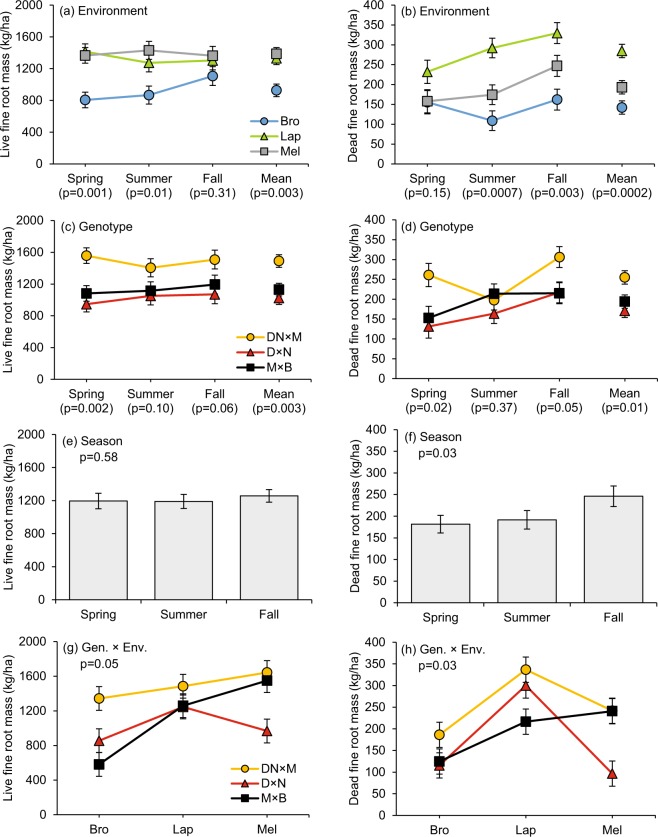
Seasonal variation in live and dead fine root mass in hybrid poplars in relation to plantation environment (**a**,**b**) and genotype (**c**,**d**). Seasonal variation in overall mean of live and dead fine root mass (**e,f**). Genotype × Environment interaction effect on (**g**) mean live fine root mass and on (**h**) mean dead fine root mass measured across the three seasons. P-value of the Environment effect (**a**,**b**), Genotype effect (**c**,**d**) and Genoytpe × Environment interaction effect (according to ANOVA) is indicated for each season and/or for across season mean. P-value of the Season effect is indicated (according to MANOVA) (**e**,**f**). Vertical bars are standard error of the mean.

Plantation environment and genotype had a significant effect on fine root biomass, but not at all sampling times (Fig. 2). The Season effect was overall not significant on live fine root biomass (p = 0.58), while it was significant on dead fine root biomass (p = 0.03), with an increase in the fall. For live and dead fine root biomass, no significant interaction was observed between the Season effect and the Genotype and/or the Environment effects (see Supplementary Table S1). The Environment effect on live fine root biomass was significant in the spring (p = 0.001), in the summer (p = 0.01), and on average across the three seasons (p = 0.003). The lowest live fine root biomass was observed at the higher fertility site (Brompton). In spring and summer, a near two-fold variation in live fine root biomass was observed across sites. Dead fine root biomass varied significantly between sites (but not in the spring), with the highest value observed at La Patrie. On average, the Genotype effect was significant on live (p = 0.003) and dead (p = 0.01) fine root biomass, with genotype DN × M having the highest biomass.

When data were averaged across the three seasons, a marginally significant Genotype × Environment interaction was observed for live (p = 0.05) and dead (p = 0.03) fine root biomass (Fig. 2g,h). However, this interaction effect was not significant for each season individually (see Supplementary Table S2). Genotype DN × M showed the smallest variation in fine root biomass across sites (1342.8–1642.6 kg/ha), while genotype M × B showed the largest (581.3–1549.0 kg/ha) (Fig. 2g).

Using data averaged across the three seasons, correlation analysis showed that many indicators of soil fertility (leaf litter N and P, soil NO_3_, P and Ca supply, base saturation, pH, CEC, clay and silt content) were significantly and negatively related to live fine root biomass across all genotypes or at the genotype level (Table 2). Conversely, indicators negatively associated to soil fertility (sand content) or positively associated to a slow rate of nutrient cycling/mineralization in the soil (leaf litter mass remaining after 1 year of incubation, C:N ratio, site elevation) were positively related to fine root biomass. No evidence of positive association between tree size and fine root biomass was observed. Dead fine root biomass was positively correlated to indicators of slow nutrient cycling/mineralization rate in the soil (NH_4_ supply, leaf litter mass remaining, site elevation, C:N ratio). However, for genotype M × B, dead fine root biomass was more strongly related to live fine root biomass, than to environmental variables.

**Table 2:** Pearson correlation coefficients (r) between live or dead fine root mass and selected abiotic and biotic factors, across all genotypes and for each genotype, in 14 year-old hybrid poplar plantations.

Factors correlated to live fine root biomass (kg/ha)	r	P-value	Factors correlated to dead fine root biomass (kg/ha)	r	P-value
**Across all genotypes (n = 27)**			**Across all genotypes (n = 27)**		
Leaf litter P (%)	−0.70	<0.0001	Soil NH_4_ supply (µg/10 cm^2^/42d)	0.72	<0.0001
Leaf litter N (%)	−0.68	0.0001	Leaf litter mass remaining after 1 yr (%)	0.67	0.0001
Soil NO_3_ supply (µg/10 cm^2^/42d)	−0.53	0.005	Site elevation (m)	0.62	0.0006
Soil base saturation (%)	−0.51	0.006	Live fine root biomass (kg/ha)	0.61	0.0008
Soil pH	−0.48	0.01	Soil pH	−0.58	0.001
Leaf litter mass remaining after 1 yr (%)	0.47	0.01	Soil CEC (meq/100 g)	−0.49	0.009
Site elevation (m)	0.47	0.01	Leaf litter N (%)	−0.49	0.010
Soil sand content (%)	0.47	0.01	Soil NO_3_ supply (µg/10 cm^2^/42d)	−0.46	0.02
Soil bulk density (g/cm^3^)	−0.46	0.01	Leaf litter C (%)	0.44	0.02
Soil CEC (meq/100 g)	−0.45	0.02	Leaf litter P (%)	−0.42	0.03
Soil C:N ratio	0.44	0.02	Soil clay content (%)	−0.42	0.03
Soil clay content (%)	−0.42	0.03	Soil stoniness (%)	0.42	0.03
Soil Ca supply (µg/10 cm^2^/42d)	−0.38	0.05	**Genotype DN × M (n = 9)**		
Soil stoniness (%)	0.38	0.05	Soil NH_4_ supply (µg/10 cm^2^/42d)	0.80	0.009
**Genotype DN × M (n = 9)**			Leaf litter mass remaining after 1 yr (%)	0.75	0.02
Soil silt content (%)	−0.77	0.02	Leaf litter Ca (%)	−0.75	0.02
Soil sand content (%)	0.70	0.04	Soil Ca supply (µg/10 cm^2^/42d)	−0.66	0.05
Soil C:N ratio	0.68	0.05	Aboveground biomass (kg/tree)	−0.66	0.05
Soil NO_3_ supply (µg/10 cm^2^/42d)	−0.67	0.05	**Genotype D × N (n = 9)**		
Aboveground biomass (kg/tree)	−0.67	0.05	Soil NH_4_ supply (µg/10 cm^2^/42d)	0.79	0.01
**Genotype D × N (n = 9)**			Soil K (µg/10 cm^2^/42d)	−0.79	0.01
Leaf litter mass remaining after 1 yr (%)	0.76	0.02	Leaf litter mass remaining after 1 yr (%)	0.79	0.01
Site elevation (m)	0.70	0.04	Site elevation (m)	0.75	0.02
**Genotype M × B (n = 9)**			Soil CEC (meq/100 g)	−0.66	0.05
Leaf litter P (%)	−0.85	0.003	**Genotype M × B (n = 9)**		
Leaf litter N (%)	−0.82	0.007	Live fine root biomass (kg/ha)	0.86	0.003
Soil sand content (%)	0.82	0.007	Soil C:N Ratio	0.78	0.01
Soil base saturation (%)	−0.76	0.02	Leaf litter N (%)	−0.76	0.02
Soil C:N ratio	0.73	0.03	Leaf litter P (%)	−0.69	0.04
Soil CEC (meq/100 g)	−0.71	0.03	Soil sand content (%)	0.67	0.05
Soil P supply (µg/10 cm^2^/42d	−0.70	0.04	Soil base saturation (%)	−0.67	0.05
Soil NO_3_ supply (µg/10 cm^2^/42d)	−0.69	0.04			
Soil pH	−0.66	0.05			
Site elevation (m)	0.66	0.05			
Soil clay content (%)	−0.66	0.05			

Only correlations with p ≤ 0.05 are shown. Mean values of live and dead fine root mass and soil nutrient supply rate (across 3 sampling times) were used in the analysis.

### Nutrient concentrations and contents of fine roots

**Figure 3:**
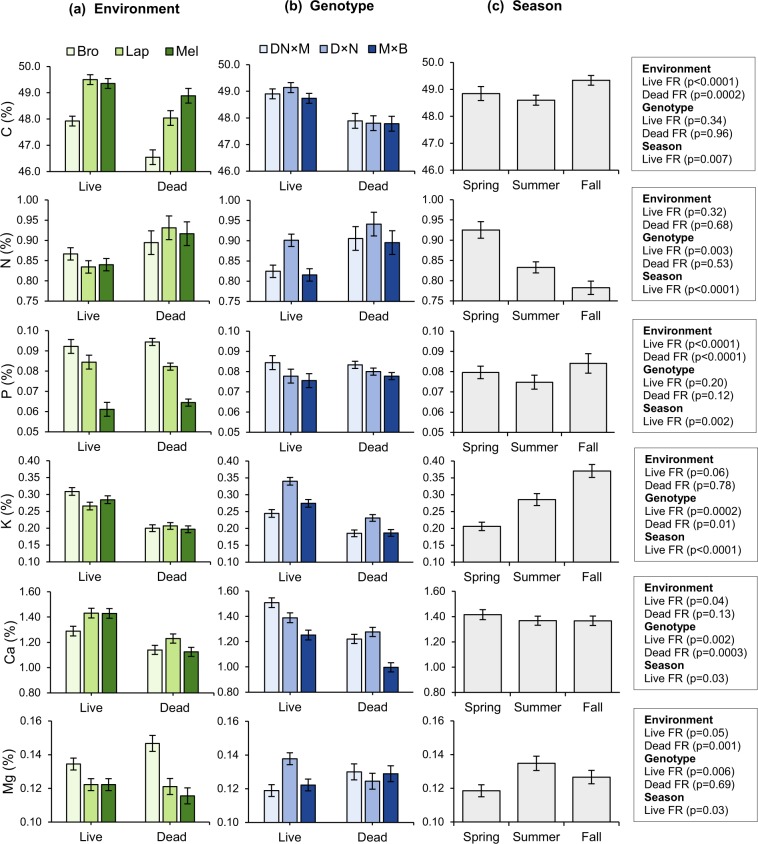
(**a**) Environment, (**b**) Genotype, and (**c**) Season effects on nutrient concentrations in fine roots (FR) of hybrid poplars. The Season effect is for live fine roots only. P-value of the Environment and Genotype effects (according to ANOVA) is indicated for both live and dead fine roots. P-value of the Season effect (live fine roots only) is indicated (according to MANOVA). Vertical bars are standard error of the mean.

Large and significant variations in fine root P concentration were observed across sites, with the smallest values observed at Melbourne, where soil P supply was the lowest (Fig. 3a, Table 1). Higher fine root Mg concentrations were also observed at Brompton, where soil Mg supply was the highest. However, no such Environment effect was observed on fine root N concentrations, despite inter-site variations in mineral N supply. Ca in live fine roots varied significantly between plantation environments, with the lowest concentration observed where soil Ca supply was the highest (Brompton). A significant Genotype effect was detected on live fine root N, K, Ca and Mg concentrations and on dead fine root K and Ca concentrations (Fig. 3b). Live fine root N, K and Mg concentrations where the highest for genotype D × N, while live fine root Ca concentration was the highest for genotype DN × M. The Season effect was significant for all nutrient concentrations in live fine roots (Fig. 3c). An important decline in live fine root N concentrations was observed from spring to fall (from 0.93% down to 0.78%), while the opposite trend was observed for K concentrations (from 0.21% up to 0.37%). There was also a significant Season × Environment interaction effect on live fine root P and K concentrations (see Supplementary Fig. S1).

At the genotype level, there were significant correlations between nutrient concentrations in fine roots and soil nutrient supplies (Table 3). Soil P was significantly correlated to live and dead fine root P concentrations for genotypes DN × M and M × B. Soil NO_3_ was significantly correlated to live and dead fine root N concentrations for genotype M × B. Significant correlations were also observed between the concentration of nutrients in live fine roots and in leaves (green foliage and litter). For genotype DN × M, nutrient concentrations in leaf litter were all significantly correlated with their respective nutrient concentrations in fine roots, except for Ca. For genotype M × B, leaf litter N and P were respectively correlated to live fine root N and P concentrations. For all genotypes, there was a significant correlation between green foliage and fine root P concentration.

**Table 3:** Pearson correlation coefficients (r) for genotype-specific correlation between soil nutrient supplies and nutrient concentrations in live or dead fine roots; and between nutrient concentrations in green foliage, or in leaf litter, and nutrient concentrations in live fine roots.

Variables	DN × M (n = 9)		D × N (n = 9)		M × B (n = 9)	
	r	P-value	r	P-value	r	P-value
**Soil nutrients supply (µg/10** **cm**^**2**^**/42 d) vs**. **Nutrient concentration (%) in live fine roots**						
Soil NO_3_ vs. Live fine roots N	0.64	0.06	−0.59	0.09	**0.84**	**0.005**
Soil P vs. Live fine roots P	**0.73**	**0.03**	0.63	0.07	**0.66**	**0.05**
Soil K vs. Live fine roots K	0.51	0.17	0.37	0.33	−0.34	0.37
Soil Ca vs. Live fine roots Ca	−0.28	0.46	0.11	0.77	−0.28	0.47
Soil Mg vs. Live fine roots Mg	0.45	0.23	−0.13	0.74	**0.75**	**0.02**
**Soil nutrients supply (µg/10** **cm**^**2**^**/42 d) vs**. **Nutrient concentration (%) in dead fine roots**						
Soil NO_3_ vs. Dead fine roots N	−0.29	0.45	−0.48	0.19	**0.71**	**0.03**
Soil P vs. Dead fine roots P	**0.68**	**0.04**	0.63	0.07	**0.79**	**0.01**
Soil K vs. Dead fine roots K	0.08	0.84	0.05	0.89	−0.27	0.47
Soil Ca vs. Dead fine roots Ca	−0.54	0.13	0.49	0.18	0.23	0.55
Soil Mg vs. Dead fine roots Mg	0.41	0.28	0.41	0.27	0.59	0.09
**Nutrient concentration (%) in green foliage vs**. **in live fine roots**						
Green foliage C vs. Live fine roots C	0.59	0.09	−0.35	0.36	**0.75**	**0.02**
Green foliage N vs. Live fine roots N	0.04	0.93	0.08	0.84	−0.03	0.93
Green foliage P vs. Live fine roots P	**0.98**	**<0.0001**	**0.72**	**0.03**	**0.82**	**0.01**
Green foliage K vs. Live fine roots K	0.09	0.82	0.28	0.46	−0.12	0.76
Green foliage Ca vs. Live fine roots Ca	−0.62	0.07	0.51	0.16	0.06	0.88
Green foliage Mg vs. Live fine roots Mg	**0.73**	**0.03**	0.41	0.28	0.62	0.07
**Nutrient concentration (%) in leaf litter vs**. **in live fine roots**						
Leaf litter C vs. Live fine roots C	**0.94**	**0.0001**	−0.51	0.16	0.35	0.36
Leaf litter N vs. Live fine roots N	**0.77**	**0.01**	−0.20	0.61	**0.66**	**0.05**
Leaf litter P vs. Live fine roots P	**0.89**	**0.001**	0.61	0.08	**0.87**	**0.002**
Leaf litter K vs. Live fine roots K	**0.73**	**0.03**	0.32	0.41	−0.07	0.85
Leaf litter Ca vs. Live fine roots Ca	−0.44	0.24	0.43	0.25	0.44	0.24
Leaf litter Mg vs. Live fine roots Mg	**0.66**	**0.05**	0.07	0.86	−0.16	0.68

Correlation coefficients in bold are significant at p ≤ 0.05. Mean values of live fine root nutrient concentration and soil nutrient supply rate (across 3 sampling periods or times) were used in the analysis.

For nutrient content in live and dead fine roots, marginally significant or non-significant Genotype × Environment interaction effects were observed for most nutrients, and only one highly significant interaction effect was observed on dead fine root Ca content (p = 0.005) (Table 4). Across sites and genotypes, live fine root nutrient content ranged 227–812 kg C/ha, 4.91–13.23 kg N/ha, 0.50–1.56 kg P/ha, 2.03–4.11 kg K/ha, 6.2–24.5 kg Ca/ha, and 0.79–1.89 kg Mg/ha. The Environment effect was highly significant on nutrient content in live and dead fine roots, except for P, K and Mg content in live fine roots (Table 4). Live fine root C, N and Ca contents were the lowest at the high fertility site of Brompton. A significant Genotype effect was also observed for C, N, P and Ca content in live and dead fine roots, with the highest values generally observed for genotype DN × M. There was also significant seasonal variation in dead fine root nutrient contents (see Supplementary Table S1), which followed the seasonal pattern of dead fine root biomass. Significant Season × Environment interaction effects were also observed for N, P and K content in live fine roots (see Supplementary Fig. S2).

**Table 4:** Nutrient stocks in live and dead fine roots of hybrid poplars in relation to genotype and plantation environment.

Effects	C (kg/ha)		N (kg/ha)		P (kg/ha)		K (kg/ha)		Ca (kg/ha)		Mg (kg/ha)	
	Live	Dead	Live	Dead	Live	Dead	Live	Dead	Live	Dead	Live	Dead
**Genotype** × **Environment**												
DN × M/Brompton	641	87.8	11.48	1.57	1.56	0.19	3.48	0.33	18.5	2.11	1.79	0.29
DN × M/La Patrie	735	163.3	11.97	3.17	1.33	0.27	3.62	0.66	24.3	4.47	1.70	0.40
DN × M/Melbourne	812	117.9	13.23	2.22	0.97	0.16	3.95	0.44	24.5	2.92	1.82	0.27
D × N/Brompton	415	53.9	7.41	1.01	0.72	0.10	3.39	0.27	11.7	1.53	1.21	0.15
D × N/La Patrie	616	144.0	11.47	2.89	1.05	0.26	3.79	0.72	17.9	4.08	1.70	0.36
D × N/Melbourne	480	47.1	8.87	0.96	0.62	0.06	3.38	0.22	13.5	1.10	1.39	0.12
M × B/Brompton	277	57.9	4.91	1.18	0.50	0.11	2.03	0.25	6.2	1.17	0.79	0.19
M × B/La Patrie	626	103.7	9.85	1.92	0.98	0.17	3.20	0.41	15.7	2.20	1.47	0.27
M × B/Melbourne	757	118.5	12.39	2.03	1.01	0.15	4.11	0.43	21.2	2.50	1.89	0.28
SE	67	14.5	1.12	0.26	0.17	0.03	0.52	0.07	1.9	0.33	0.22	0.04
P-value	0.06	0.03	0.05	0.02	0.06	0.06	0.38	0.03	0.04	0.005	0.15	0.13
**Environment**												
Brompton	444	66.5	7.93	1.26	0.93	0.14	2.97	0.28	12.1	1.60	1.26	0.21
La Patrie	659	137.0	11.09	2.66	1.12	0.23	3.54	0.60	19.3	3.58	1.63	0.34
Melbourne	683	94.5	11.50	1.74	0.87	0.13	3.81	0.36	19.7	2.17	1.70	0.22
SE	39	8.4	0.65	0.15	0.10	0.02	0.30	0.04	1.1	0.19	0.13	0.02
P-value	0.002	0.0003	0.004	<0.0001	0.20	0.0005	0.17	0.0002	0.0005	<0.0001	0.07	0.004
**Genotype**												
DN × M	729	123.0	12.23	2.32	1.29	0.21	3.68	0.48	22.4	3.17	1.77	0.32
D × N	504	81.7	9.25	1.62	0.80	0.14	3.52	0.40	14.3	2.24	1.44	0.21
M × B	553	93.3	9.05	1.71	0.83	0.15	3.11	0.36	14.4	1.95	1.38	0.24
SE	39	8.4	0.65	0.15	0.10	0.02	0.30	0.04	1.1	0.19	0.13	0.02
P-value	0.004	0.01	0.007	0.01	0.007	0.02	0.42	0.15	0.0002	0.002	0.11	0.02

Mean values of live fine root nutrient contents (across 3 sampling times) were used in the ANOVA (SE = Standard error of the mean).

N content of live fine root biomass was generally correlated to the same factors observed for live fine root biomass, as those two variables were strongly correlated (r = 0.88–0.99, depending on the genotype) (Tables 2 and 5). For, genotype DN × M, soil variables were not significantly correlated to N content in live fine roots, which varied little between sites (Table 4). Correlations between live fine root biomass and P content were found for genotypes D × N and M × B, but these were weaker compared to correlations observed with N content (Table 5). For genotype DN × M, live fine root biomass and P content were not significantly correlated, and live fine root P content was significantly and positively correlated to several soil fertility indicators. Correlations between soil fertility indicators and live fine root P content were also positive for genotype D × N, but negative for genotype M × B.

**Table 5:** Pearson correlation coefficients (r) between N or P content in live fine root mass and selected abiotic and biotic factors, across all genotypes and for each genotype, in 14 year-old hybrid poplar plantations.

Factors correlated to N content (kg/ha) in live fine root biomass	r	P-value	Factors correlated to P content (kg/ha) in live fine root biomass	r	P-value
**Across all genotypes (n = 27)**			**Across all genotypes (n = 27)**		
Live fine root mass (kg/ha)	0.97	<0.0001	Live fine root mass (kg/ha)	0.74	<0.0001
Leaf litter P (%)	−0.62	0.0005	Leaf litter N (%)	−0.53	0.005
Leaf Litter N (%)	−0.57	0.002	Leaf litter P (%)	−0.40	0.04
Soil base saturation (%)	−0.50	0.008	**Genotype DN** × **M (n** = **9)**		
Soil CEC (meq/100 g)	−0.50	0.008	Soil clay content (%)	0.83	0.006
Soil NO_3_ supply (µg/10 cm^2^/42d)	−0.49	0.009	Soil Ca supply (µg/10 cm^2^/42d)	0.71	0.03
Leaf litter mass remaining after 1 yr (%)	0.48	0.01	Soil P supply (µg/10 cm^2^/42d)	0.70	0.04
Site elevation (m)	0.47	0.01	Leaf litter P (%)	0.70	0.04
Soil clay content (%)	−0.46	0.01	**Genotype D** × **N (n** = **9)**		
Soil bulk density (g/cm^3^)	−0.46	0.02	Live fine root mass (kg/ha)	0.85	0.004
Soil sand content (%)	0.46	0.02	Soil silt content (%)	0.75	0.02
Soil pH	−0.46	0.02	Soil sand content (%)	−0.68	0.04
Soil C:N ratio	0.43	0.02	**Genotype M × B (n = 9)**		
**Genotype DN × M (n = 9)**			Live fine root mass (kg/ha)	0.95	0.0001
Live fine root mass (kg/ha)	0.88	0.002	Leaf litter N (%)	−0.77	0.02
**Genotype D × N (n = 9)**			Soil CEC (meq/100 g)	−0.77	0.02
Live fine root mass (kg/ha)	0.98	<0.0001	Soil pH	−0.77	0.02
Leaf litter mass remaining after 1 yr (%)	0.79	0.01	Soil base saturation (%)	−0.73	0.03
Site elevation (m)	0.76	0.02	Soil NO_3_ supply (µg/10 cm^2^/42d)	−0.70	0.04
Soil NO_3_ supply (µg/10 cm^2^/42d)	−0.66	0.05	Soil NH_4_ supply (µg/10 cm^2^/42d)	0.70	0.04
**Genotype M × B (n = 9)**			Site elevation (m)	0.67	0.05
Live fine root mass (kg/ha)	0.99	<0.0001	Leaf litter P (%)	−0.67	0.05
Leaf litter P (%)	−0.85	0.004	Soil sand content (%)	0.66	0.05
Soil sand content (%)	0.82	0.007			
Leaf litter N (%)	−0.80	0.009			
Soil base saturation (%)	−0.74	0.02			
Soil C:N ratio	0.72	0.03			
Soil CEC (meq/100 g)	−0.71	0.03			
Leaf litter Ca (%)	0.71	0.03			
Soil clay content (%)	−0.69	0.04			
Soil P supply (µg/10 cm^2^/42d)	−0.67	0.05			
Soil NO_3_ (µg/10 cm^2^/42d)	−0.66	0.05			

Only correlations with p ≤ 0.05 are shown. Mean values of N and P content in live fine root mass and of soil nutrient supply rate (across 3 sampling periods) were used in the analysis.

## Discussion

Previous field studies with planted poplars have reported different conclusions related to the effect of abiotic and biotic factors on fine root biomass^35–38,40,41^. Our regional-scale study, conducted in 14 year-old closed-canopy poplar plantations, provides evidence supporting the negative relationship hypothesis between soil fertility and fine root biomass^13^ (Fig. 2a, Table 2). Live fine root biomass was generally the lowest at the high fertility site (Brompton) (Table 1, Fig. 2a). Moreover, the general and genotype-specific correlation analysis shows that fine root biomass was negatively correlated to several indicators of soil fertility in the mineral layer (supply of NO_3_, P and Ca, base saturation, pH, CEC, clay and silt content) and in the organic layer (leaf litter N and P concentrations), while being positively correlated to indicators of low soil fertility (sand content) and of slower soil nutrient cycling rate (C:N ratio, leaf litter mass remaining, site elevation) (Table 2). However, we found no evidence of positive relationships between aboveground biomass and fine root biomass, despite the wide range of tree sizes that was sampled (ranging 144.8–223.2 kg/tree for DN × M, 100.5–193.6 kg/tree for M × B, and 39.9–153.6 kg/tree for D × N). Such a result contrasts with observations from younger poplar plantations and forest stands^10,18,20,37^, and suggests that fine root biomass and aboveground growth are uncoupled in closed-canopy stands^18^.

In our study, variations in soil NO_3_ supply, within and between sites, possibly influenced fine root biomass of hybrid poplars. As outlined by Aber *et al*.^56^, NO_3_ is much more mobile than NH_4_ in the soil, potentially reducing the need for trees to maintain high fine root biomass under high NO_3_ availability. Overall, we observed the lowest live fine root biomass at Brompton, where NO_3_ was highly available and the dominant N-form in the soil (Table 1, Fig. 1a). Seasonal results from this site further suggest that the steep soil NO_3_ decline during the growing season led to a positive feedback on live fine root biomass in the fall (Figs 1a and 2a). Yet, at the lower fertility and colder sites (Melbourne and La Patrie), NO_3_ supply remained low during the growing season (Fig. 1a), potentially leading to high and fairly constant live fine root biomass from spring to fall (Fig. 2a). Such results are consistent with the root growth modulation mechanism in response to N supply, previously shown for young hybrid poplars^39^. From an evolutionary perspective, plasticity in fine root growth in response to variations in soil resource availability likely reflects the adaptation of poplars (*Tacamahaca* and *Aigeiros* sections) to riparian environments, where water availability and soil N supply fluctuates widely during the growing season in relation to hydrology^57,58^. Such an increase in root foraging capacity on lower fertility sites could have allowed the studied genotypes to maintain relatively stable aboveground biomass yields across sites (Table 1). Previous results from the same experimental design have also shown that leaf N and P resorption proficiency (*i*.*e*. extent to which nutrient concentrations have been reduced in dead leaves^59^) increased with declining soil fertility^49^. Thus, nutrient conservation strategy in the canopy and belowground resource uptake strategy appeared to be coupled, and controlled by site fertility in mature hybrid poplar plantations.

Variations in dead fine root biomass were mainly driven by the plantation environment (Fig. 2). The highest dead fine root biomass was observed at the higher elevation sites (lowest MAT) (Table 1, Fig. 2b), as colder temperatures generally slow the rate of organic matter decay^60^, thus providing favorable conditions for the accumulation of dead roots in the soil. Accordingly, we observed strong positive correlations between dead fine root biomass and indicators of reduced organic matter mineralization rate (site elevation, leaf litter mass remaining, soil NH_4_ and C:N ratio) (Table 2). The higher fine root biomass on high elevation and less fertile sites also contributed to maintaining high dead fine root biomass in the soil (Table 2, Fig. 2). For that reason, negative correlations between soil fertility indicators and dead fine root biomass were also observed (Table 2).

In agreement with several studies^24–28^, fine root biomass and its plasticity level substantially differed between genotypes (Fig. 2g). Thus, the environmental gradient did not affect fine root biomass of the different genotypes with the same magnitude. Interestingly, genotype DN × M, which had low plasticity and high fine root biomass, and genotype M × B, which had large plasticity in fine root biomass, reached the highest aboveground biomass yields across sites (Table 1). Low-yielding genotype D × N is known for its greater dependency on the nutrient mineralization pathway because it is less proficient at resorbing N, P and K from foliage^49^ (Table 1). This could explain why leaf litter mass remaining was the strongest factor related to its fine root biomass (Table 2).

Genotype DN × M, which produced low quality and more recalcitrant leaf litter, had the highest fine root mass overall (Fig. 2c,d, Table 1). This supports the compensatory root growth hypothesis^29^, although no negative feedback of this genotype was observed on soil N supplies. By having a high fine root biomass with high Ca concentration, root tissues of genotype DN × M (Figs 2 and 3) potentially buffered the soil against the negative impacts of its low leaf litter quality. In deciduous trees, fine roots rich in Ca tend to have higher decay rates, suggesting that Ca-rich roots have a positive feedback on decomposition processes in the soil^61,62^. There was also evidence for plasticity in live fine root Ca concentrations, with highest values observed at Melbourne and La Patrie sites, where soil Ca supplies, pH and overall fertility were the lowest (Table 1). Moreover, C, N, P, K, and Mg concentrations observed in live fine roots of hybrid poplars were in the range of mean values observed in global data sets^9,62^, but Ca concentrations (ranging 1.29–1.43% across sites) were much higher than the reported average for broadleaved trees (0.21%)^62^. Clearly, the ecological significance of these observations deserves further investigation given the key role of Ca input from trees in pedogenesis^63^.

As expected, genotype D × N had the highest concentrations of N and K in live fine roots, which reflects its higher N and K concentrations in foliage and leaf litter (Table 1)^49^. However, fine root P concentrations was little affected by the genotype, which contrasts with the large variations in foliage and leaf litter P concentrations previously observed (Table 1)^49^. Moreover, Ca rich leaf litter of genotype M × B was not reflected in fine root chemistry (Table 1, Fig. 3b). These observations suggest that nutrient concentrations in foliage or leaf litter are only partly reflected in the fine roots of the studied genotypes.

While fine root biomass was inversely correlated to indicators of soil fertility, fine root nutrient concentrations were generally positively correlated to their respective supply in the mineral soil, or their concentration in the organic soil layer (i.e. leaf litter), or in green foliage. While such covariation pattern was expected, it was more evident for genotypes DN × M and M × B, and appeared to be especially strong for P and N. Surprisingly, the large inter-site variations in soil N supply were little reflected in fine root N concentrations (Table 1, Fig. 3a), as seen in another study with *P*. *tremuloides*^36^. This suggests potentially stronger P-limitations than N-limitations in soils of the study area^64^. Consequently, fine root biomass more strongly predicted fine root N content than P content (Table 5). We even observed no significant correlation between fine root biomass and P content for genotype DN × M. Because fine root P concentration of all genotypes was positively affected by the fertility gradient (Fig. 3a, Table 3), genotypes exhibiting lower plasticity in fine root biomass (i.e. DN × M and D × N) had their fine root P content positively associated to soil fertility indicators (Fig. 2g, Table 5). Thus, for certain genotypes, site fertility can have a negative effect on fine root biomass, but a positive effect on the nutrient pool it contains (Tables 2 and 5). Similarly, the relationships of fine root biomass and nutrient content with climatic variables led to opposite trends across boreal forests^13^.

Strong seasonal trends were also observed on both fine root biomass and nutrients. However, the hypothesis that greater live fine root biomass would be observed in the spring during canopy growth^40,46^ was not supported by the data. Such seasonal peak may be more characteristic of younger poplar plantations^37^. In mature trees, the source of N needed to fuel canopy growth comes primarily from the remobilization of internal N reserves^65^, reducing the need for root expansion in the spring, especially if soil N supplies are high (Fig. 1a,b). As hypothesized, dead fine root biomass peaked in the fall (Fig. 2f), bringing additional evidence that fine root mortality increases during leaf senescence^46^. The same seasonal effect occurred for nutrient content in dead fine roots (see supplementary Table S1), as dead fine root nutrient concentrations were only measured on composite samples combining root material from the three sampling times. Fine root N and K concentrations were also subjected to strong, but opposite, seasonal variations (Fig. 3c). In 1-year-old poplars, Pregitzer *et al*.^26^ observed increases in fine root N concentrations in fall, suggesting that a fraction of N resorbed from foliage was stored in these roots. Yet, we observed a decrease in fine root N concentration from spring to fall, which appeared to be related to the seasonal decline in soil N supply (Fig. 1a,b). Concurrently, this N decline could be partly related to N resorption, although evidence of such nutrient conservation mechanism remains controversial in root tissues^21,66,67^. The role of fine roots as storage and/or resorption sites for assimilated N, and its relationship to ontogeny and rooting order remains to be clarified. Contrary to the pattern observed for fine root N, we observed an increase in fine root K concentration from spring to fall. This change in root K was likely related to the seasonal increase in soil K supply (Figs 2d and 3c), as K leaching from poplar stand canopy peaks during leaf senescence^68^.

Estimates from the boreal forest showed that fine root biomass and N content of *Populus* stands respectively averaged 4800 kg/ha and 46.7 kg N/ha in the 0–20 cm soil layer^13^. This is well above the measured range for live fine root biomass (581.3–1642.6 kg/ha) and N content (4.91–13.23 kg N/ha), which corresponds to observations from other poplar plantations in the temperate zone^37,38^. Such high fine root biomass in boreal poplar stands vs. temperate old-field plantations likely reflects climate-related limitations in soil resource availability and in nutrient uptake rate by roots as latitude increases^46^. The positive effect of agricultural legacies (i.e. fertilization, liming, soil cultivation, pastoralism, legume cover crops) on plantation soil fertility could also have contributed in reducing the necessity for trees to maintain high fine root biomass. The core method, which is widely used, also tends to overestimate fine root biomass compared to the excavation method we used^69^.

In conclusion, this study has shown that fine root biomass, chemistry, and elemental content of mature poplars are under strong environmental and genetic control, and that seasonal variations in some these traits also occur. It was difficult to isolate a single soil or climatic factor driving changes in fine root biomass across sites, as these factors tend to be correlated in the study area^6,49^. Furthermore, along the studied gradient, the plasticity level observed for some traits (fine root biomass, C, N, P and Ca content) was genotype-specific and high for some genotypes. Consequently, it will be challenging to quantify the elemental pools located in fine roots of natural and novel ecosystems dominated by poplars, and to predict the effects of global environmental changes on these pools, especially considering (1) the hundreds of species, subspecies, hybrids and cultivars within the *Populus* genus, and (2) the wide climatic, edaphic and topographic gradients along which poplars are naturally distributed and planted^70^. Such uncertainties need to be considered in biogeochemical models, in ecosystem services assessments and for long-term site productivity management. For the stand type studied, fine root biomass and elemental content were also poorly correlated to aboveground biomass, suggesting that these ecosystem properties are unlikely to be accurately predicted from forest inventory and airborne LiDAR data. Field studies evaluating fine root/environment relationships across large resource and climatic gradients, and involving different poplar species and stand ages, are needed to address these challenges^71^.

From a management perspective, our study pointed out that some poplar genotypes maintain a high fine root biomass across edaphic/climatic gradients. This may be a desirable root trait for environmental applications (*i*.*e*. phytoremediation, erosion control, soil restoration, C storage belowground). Finally, the small aboveground productivity differences observed across the studied gradient suggest that plasticity in fine root biomass growth may contribute to overcome nutrient limitations that often characterize marginal agricultural lands targeted for afforestation. Therefore, old-field sites located at higher elevation (colder climate) and characterized by moderate soil fertility could represent the best opportunities to simultaneously increase wood production and store C belowground with fast-growing poplars, providing that appropriate genotypes are selected.

## Methods

### Plantation sites and experimental design

In 2013, three plantations of 14 year-old poplars were selected to evaluate how plantation environment, genotype and season affect various fine root traits in closed-canopy stands. These plantation were established on old-field sites in the Estrie region of the province of Québec (Southeastern Canada). The names of the study sites are names of cities or towns near which a plantation was established in 2000: Brompton (Bro), La Patrie (Lap) and Melbourne (Mel). All situated within a 40 km radius, the study sites were selected from a larger network of *Populus* plantations because of their contrasted edaphic characteristics and position along a regional elevation gradient (from 170 m up to 440 m a.s.l.)^6^ (Table 1). Lower MAT and MAP characterize higher elevation sites regionally (Table 1)^72^. For each site, 30-years average climatic data (1981–2010)^73^ were taken from the nearest meteorological station (always located within a 25 km radius of a site and at similar elevation). Prior to plantation establishment, the three old-field sites were dominated by an herbaceous vegetation cover. Additional details about plantation site characteristics, site preparation and tending operations can be found in previous studies^6^.

At each plantation site, a randomized block design was established, with 3 blocks (nested in sites) and 3 plots per block (one per genotype), for a total of 27 experimental plots (3 sites × 3 blocks × 3 genotypes, n = 27). Each plot was 12 × 12 m and initially contained 12 trees (from the same genotype) planted with 3 m × 4 m spacing for a planting density of 833 trees/ha. The three genotypes selected for this study had different parentages: (1) D × N-131 a *P*. *deltoides* × *P*. *nigra* hybrid (also named *P*. × *canadensis*); (2) DN × M-915508, a *P*. *canadensis* × *maximowiczii* hybrid; and (3) M × B-915311, a *P*. *maximowiczii* × *balsamifera* hybrid. Developed in Québec by the Ministère des Forêts, de la Faune et des Parcs (MFFP), these genotypes showed superior disease resistance and growth traits in genetic selection tests undertaken in the study area^74^.

### Mineral soil characteristics

At the plot-level, two soil cores (inner corer diameter of 5.2 cm) were extracted from the 0–20 cm surface layer (without the litter layer) to form a composite soil sample. Soil samples were air dried. Following sieving (mesh size = 2 mm), air-dry mass of each soil sample was recorded and a subsample was taken to determine an oven-dry mass (105 °C) to air-dry mass ratio, to calculate dry mass of soil samples. Soil bulk density of the fine earth fraction was calculated by dividing the dry mass of the fine earth fraction by the volume of soil cores^75^. Coarse fragments (i.e. stones with diameter > 2 mm) were weighted and their volume was estimated assuming a density of 2.65 g/cm^2^ ^76^. Stoniness was calculated by dividing coarse fragment volume by the soil volume extracted with cores.

The methods used for C and N concentration determination in soil and for basic soil analyses have all been described in earlier studies^30,49^. The dynamics of soil nutrients (NO_3_, NH_4_, P, K, Ca, and Mg) in the 0–10 soil layers was evaluated with the Plant Root Simulator (PRS^TM^-Probes) technology (Western Ag Innovations Inc., Saskatoon, SK, Canada), a type of ion exchange membrane. At the plot level, a composite of four pairs of probes (each pair has a cationic and an anionic probe) were inserted into the soil for three consecutive time periods of 42 days in 2013: (1) May 16/June 27 (*i*.*e*. late spring), (2) June 27/August 8 (*i*.*e*. early summer), and (3) August 8/September 19 (*i*.*e*. late summer)^49^. Overall, 81 PRS-probes samples were collected (27 plots × 3 sampling periods).

### Fine root sampling, chemical analysis and nutrient content calculations

Fine root biomass was sampled in 14-year old poplar plantations using pit excavations^69^ at three different sampling times during the growing season: (1) in late May (27–29 May, 2013), in late July (22–24 July 2013) and in late October (21–23 October, 2013), which correspond to important periods in the annual growth cycle of hybrid poplars^77^. These sampling times are referred as spring (late May), summer (late July) and fall (late October) in Figures. In each plot and for each sampling time, one pit of 25 × 50 cm in area by 20 cm depth (soil volume = 25,000 cm^3^) was excavated near a representative healthy tree (of average size in the plot). A total of 81 pits were excavated (3 sites × 3 blocks/site × 3 genotypes/block × 3 sampling times). Pits were located 75 cm away from the tree base towards the center of the inter-row space. A 25 × 50 cm cutting guide and spray paint were used to properly delimit the sampling area on the soil surface. All soil and roots extracted from a single pit were placed on a large tarp and roots were separated from the soil manually. Poplar roots were separated from roots of understory vegetation roots (mostly herbaceous plants and ferns), based on visual criteria (i.e. colour and morphology). Each poplar root sample was placed in a sealed plastic bag and kept frozen (−10 °C) until it could be processed. Root samples were then washed and only fine roots (diameter <2 mm) were selected using a digital caliper. Fine roots were separated into two categories; live fine roots and dead fine roots. This separation was based on root colour and elasticity, with live roots being pale brown and elastic, and dead roots being dark brown or black, and easy to break^25,78^. Living roots were also characterised by a better cohesion between the cortex and the periderm^25^. Clean fine roots samples were then oven-dried (60 °C) to constant mass to determine their dry mass. Live and dead fine root mass samples were scaled to per ha basis for comparison with other studies.

The methods used for elemental concentration determination in plant tissues (live and dead fine roots) have been described in an earlier study^49^. For chemical analyses of dead fine roots, a composite sample was made at the plot-level by combining equal mass from root samples collected over the three sampling times (total of 27 samples). However, for chemical analysis on live fine roots, samples collected at each of three different times where used in each plot (total of 81 samples). Carbon and nutrient content in fine roots were calculated in each plot and for each of the three sampling times. For live fine roots, elemental concentrations obtained from each of the three sampling times were respectively multiplied by live root mass measured at each of the three sampling times. For dead fine roots, mean elemental concentrations measured across the three sampling times were multiplied by dead fine root mass measured at each of the three sampling times.

### Aboveground woody biomass of sampled trees

The diameter at breast height (DBH) of each tree sampled for fine roots was recorded at the end of the 14^th^ growing season. Aboveground woody biomass of these trees was calculated with hybrid-specific allometric relationships previously developed with 13 year-old hybrid poplars from a larger plantation network that included the three sites of this study^79^.

### Statistical analyses

For data collected once in each plot or for data averaged across the three sampling times, a two-way ANOVA in a fixed factorial design was used to test the main effects (Environment and Genotype) and the interaction effect (Environment × Genotype). For repeated measures data collected at the plot-level at three different times or periods during the growing season (i.e. soil nutrient supply rates, live and dead fine root mass, nutrient concentrations in live fine roots, and nutrient content in live and dead fine roots) a multivariate analysis of variance (MANOVA) was used to test for the Season factor, its interactions with other main effects, and with the interaction effect (*i*.*e*. Environment, Genotype and Environment × Genotype). Pillai’s trace test-statistic was used to declare significant interaction effects (Season × Environment; Season × Genotype; Season × Environment × Genotype), while the F-test was used to declare significant Season effects. Following ANOVA or MANOVA, the normality of residuals distribution was verified using the Shapiro-Wilk W-test. Only soil NO_3_ supply rate data had to be ln (y + 1) transformed to meet the assumption of normality in residuals distribution. Finally, the Pearson product-moment correlation coefficient (*r*) was used to measure the strength of linear relationships between environmental variables and root traits, or between root traits. Data related to elemental concentrations of green foliage and of leaf litter (collected in 2012), and data from a leaf litter decay experiment done in 2013, all from the same experimental design^49^, were included in the correlation analyses. All statistical analyses were done using JMP (version 11) from SAS Institute (Cary, NC, United States).

## Supplementary information

Supplementary Tables and Figures

## Data Availability

The dataset collected and analysed during the current study is available from the corresponding author upon reasonable request.
