# Supplementary material for: Abiotic and biotic factors controlling fine root biomass, carbon and nutrients in closed-canopy hybrid poplar stands on post-agricultural land
Source: Sci Rep. 2019 Apr 18;9:6296. doi: 10.1038/s41598-019-42709-6 (PMC6472364; doi:10.1038/s41598-019-42709-6)

Supplementary files

Abiotic and biotic factors controlling fine root biomass, carbon and nutrients in closed-canopy hybrid poplar stands on post-agricultural land. Julien Fortier, Benoit Truax, Daniel Gagnon and France Lambert.

Table S1. P-values for the Season effect and interaction effects (according to MANOVA) on fine root and soil variables measured during the growing season in hybrid poplar plantations.

**Variables**	**Season**	**Season × Environment**	**Season × Genotype**	**Season × Genotype × Environment**
**Fine root biomass (kg/ha)**				
Live fine root biomass	0.58	0.24	0.78	0.53
Dead fine root biomass	**0.03**	0.35	0.13	0.98
**Live Fine root nutrient concentration (%)**				
C	**0.007**	0.48	0.81	0.85
N	**<0.0001**	0.14	0.44	0.70
P	**0.002**	**0.001**	0.68	0.22
K	**<0.0001**	**<0.0001**	0.10	0.55
Ca	**0.03**	0.17	0.55	0.36
Mg	**0.03**	0.21	0.86	0.51
**Live fine root nutrient content (kg/ha)**				
C	0.46	0.26	0.81	0.52
N	0.20	**0.03**	0.76	0.36
P	0.16	**0.05**	0.90	0.84
K	**0.0006**	**0.002**	0.47	0.88
Ca	0.54	0.38	0.77	0.55
Mg	0.36	0.13	0.90	0.43
**Dead fine root nutrient content (kg/ha)**				
C	**0.03**	0.35	0.13	0.98
N	**0.03**	0.36	0.15	0.98
P	**0.04**	0.37	0.12	0.96
K	**0.03**	0.44	0.19	0.99
Ca	**0.03**	0.39	0.12	0.98
Mg	**0.04**	0.34	0.11	0.98
**Soil nutrient supply rate (µg/10cm^2/42d)**				
NO_3 (ln transformed)	**<0.0001**	**0.05**	0.57	0.64
NH_4	**0.0005**	**0.01**	0.34	0.43
P	**0.02**	0.15	0.6	0.26
K	**0.004**	0.21	**0.05**	0.58
Ca	**0.03**	0.65	0.62	0.37
Mg	**0.03**	**0.03**	0.07	0.11

Table S2. P-value for the main effects (Environment and Genotype) and interaction effects (according to two-way ANOVA) on fine root and soil variables measured during the growing season in hybrid poplar plantations.

**Variables**	**Environment**	**Genotype**	**Genotype × Environment**
**Fine root biomass (kg/ha)**			
Live fine root biomass (mean across 3 seasons)	**0.003**	**0.003**	**0.05**
Live fine root biomass (spring)	**0.001**	**0.002**	0.06
Live fine root biomass (summer)	**0.01**	0.10	0.11
Live fine root biomass (fall)	0.31	0.06	0.42
Dead fine root biomass (mean across 3 seasons)	**0.0002**	**0.01**	**0.03**
Dead fine root biomass (spring)	0.15	**0.02**	0.57
Dead fine root biomass (summer)	**0.0007**	0.37	0.18
Dead fine root biomass (fall)	**0.003**	**0.05**	0.08
**Live fine root nutrient concentration (%)**			
C	**<0.0001**	0.34	0.30
N	0.32	**0.003**	0.14
P	**<0.0001**	0.20	0.07
K	0.06	**0.0002**	0.26
Ca	**0.04**	**0.002**	0.20
Mg	**0.05**	**0.006**	0.53
**Dead fine root nutrient concentration (%)**			
C	**0.0002**	0.96	0.74
N	0.68	0.53	0.27
P	**<0.0001**	0.12	0.07
K	0.78	**0.01**	0.90
Ca	0.13	**0.0003**	0.19
Mg	**0.001**	0.69	0.17
**Live fine root nutrient content (kg/ha)**			
C	**0.002**	**0.004**	0.06
N	**0.004**	**0.007**	**0.05**
P	0.20	**0.007**	0.06
K	0.17	0.42	0.38
Ca	**0.0005**	**0.0002**	**0.04**
Mg	0.07	0.11	0.15
**Dead fine root nutrient content (kg/ha)**			
C	**0.0003**	**0.01**	**0.03**
N	**<0.0001**	**0.01**	**0.02**
P	**0.0005**	**0.02**	0.06
K	**0.0002**	0.15	**0.03**
Ca	**<0.0001**	**0.002**	**0.005**
Mg	**0.004**	**0.02**	0.13
**Aboveground woody biomass**			
Sampled trees for fine root biomass (kg/tree)	0.41	**0.0001**	0.56
Stand level (t/ha)	0.17	**0.002**	0.66
**Soil nutrient supply rate (µg/10cm^2/42d)**			
NO_3 (ln transformed)	**<0.0001**	0.61	0.88
NH_4	**0.0003**	0.25	0.75
P	**0.007**	0.47	0.57
K	**0.05**	0.36	0.28
Ca	**0.01**	0.56	0.43
Mg	**0.0002**	0.76	0.79
**Soil characteristics**			
Bulk density (g/cm^3)	**<0.0001**	0.71	0.96
pH	**<0.0001**	0.63	0.57
Organic matter (%)	**<0.0001**	0.51	0.13
C (mg/g)	**0.0005**	0.76	0.77
N (mg/g)	**0.009**	0.44	0.55
C/N	**<0.0001**	0.45	0.84
CEC (meq/100g)	**0.01**	0.91	0.91
Base saturation (%)	**<0.0001**	0.81	0.58

Figure S1. Season × Environment interaction effect on live fine root P ($p=0.001$) and K ($p<0.0001$) concentrations in hybrid poplar plantations.

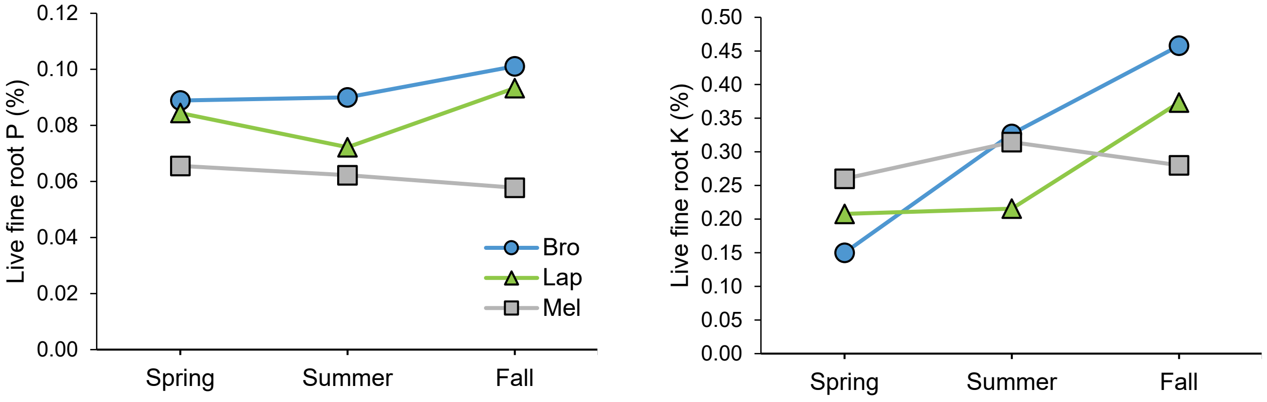

Figure S2. Season × Environment interaction effect on live fine root N ($p=0.03$), P ($p=0.05$) and K ($p=0.002$) contents in hybrid poplar plantations.

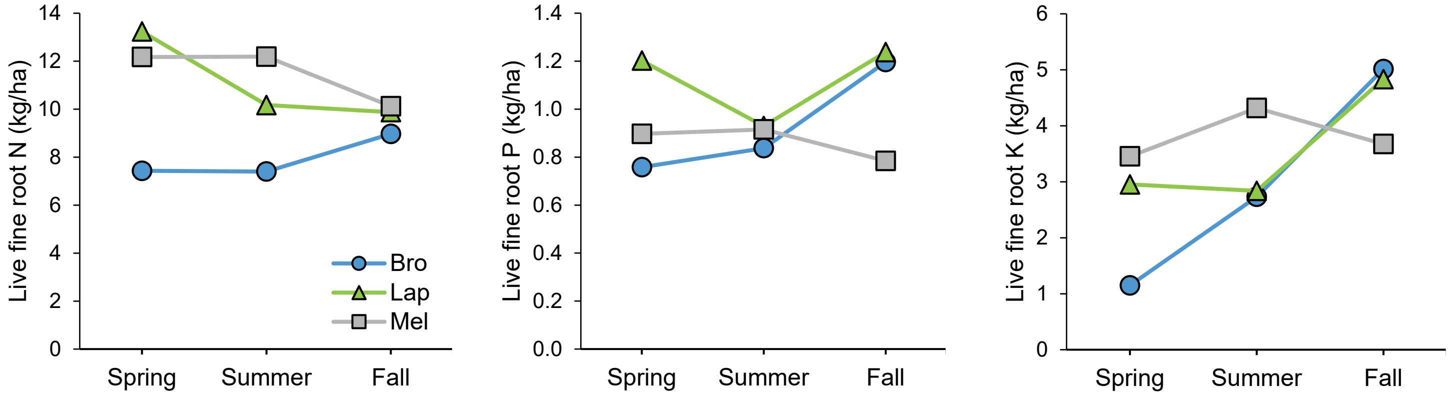

Supplement: Supplementary file 1 — Supplementary Tables and Figures [file 41598_2019_42709_MOESM1_ESM.pdf]
